# Methotrexate significantly reduces the humoral vaccination response against SARS-CoV-2 in older but not younger patients with rheumatoid arthritis

**DOI:** 10.1007/s00296-022-05123-2

**Published:** 2022-04-16

**Authors:** Martin Feuchtenberger, Magdolna Szilvia Kovacs, Anna Eder, Axel Nigg, Arne Schäfer

**Affiliations:** 1 MED|BAYERN OST Medizinische Versorgungszentren Altötting Burghausen, Krankenhausstraße 1, 84489 Burghausen, Germany; 2 Medizinische Klinik und Poliklinik II, Klinikum der Universität Würzburg, Würzburg, Germany; 3 Diabetes Zentrum Mergentheim, Bad Mergentheim, Germany

**Keywords:** Methotrexate, Arthritis, Rheumatoid, SARS-CoV-2, COVID-19, Vaccination, Immune response

## Abstract

To assess the humoral response to vaccination against SARS-CoV-2 in patients with rheumatoid arthritis treated with methotrexate (MTX). In total, 142 fully vaccinated individuals were included at 6 ± 1 weeks after their second vaccination [BioNTech/Pfizer (70.4%), AstraZeneca (20.4%), and Moderna (9.2%)]. The primary goal was to assess the humoral immune response as measured by titres of neutralising antibodies against the S1 antigen of SARS-CoV-2. In a cross-sectional, single-centre study, titres were compared between patient subgroups with (*n* = 80) and without (*n* = 62) methotrexate exposure. MTX patients showed a significantly reduced humoral response to vaccination in the oldest patient subgroup (> 70 years: *P* = 0.038), whereas titres of neutralising antibodies were not significantly different between MTX and non-MTX patients in patients less than 70 years of age (< 56 years: *P* = 0.234; 56–70 years: *P* = 0.446). In patients > 70 years, non-MTX patients showed a maximum immune response in 76.5% of cases, whereas this percentage was reduced to 53.7% in study participants on MTX medication (effect size *d* = 0.21). Older age in patients with rheumatoid arthritis in combination with methotrexate results in a significantly reduced humoral response after vaccination against SARS-CoV-2. Our data underline the importance of age regarding the humoral response and may support the temporary cessation of methotrexate, particularly in elderly patients in the context of vaccination against SARS-CoV-2.

## Introduction

Vaccination against SARS-CoV-2 is considered the most effective and definitive measure to prevent infection and reduce morbidity and mortality in cases of infection. Very early on, the question arose as to whether patients with inflammatory rheumatic disease differ from the normal population in terms of disease risk, the course of the infection, or response to vaccination against SARS-CoV-2 [1–5]. Data on the immunogenicity of COVID-19 vaccines in autoimmune diseases are lacking from the original vaccine trials leading to vaccination approval, especially since these patients were excluded to a large extent from the trials [6, 7]. The question also arose early on as to how the success of vaccination should be validly measured. In this context, in addition to the humoral response in the form of neutralising antibodies measured in binding antibody units (BAU), the formation of a T cell response must also be considered [8]. However, this still does not clarify the clinical implications of such results. None of the current studies have prospectively investigated the clinical endpoint of infection prevention by vaccination in a large rheumatic collective. However, the literature available on laboratory-based endpoints for vaccination success also comes to partly inconsistent results concerning immunogenicity measured by neutralising antibodies or T cell-based immune responses [9]. The first studies identified additional factors, such as underlying disease, age, co-medication (e.g. with glucocorticoids) or comorbidities, in addition to the immunomodulatory or immunosuppressive therapies used at the time of vaccination. The fact that vaccination response in patients with inflammatory rheumatic diseases is influenced by additional, numerous factors have been known for quite a long time from vaccination studies against pneumococcus or influenza [10]. Our study aimed to compare the extent of the humoral response to a two-dose vaccination against SARS-CoV-2 between patients with rheumatoid arthritis (RA) under methotrexate (MTX) and a reference group including subjects not suffering from RA or receiving MTX. The immune response was measured by the titres of neutralising antibodies to the spike protein under normal clinical conditions. In addition to MTX, we investigated the influence of patient age as—in addition to methotrexate—the putatively most relevant factor for the humoral vaccination response in this study context.

## Methods

### Study participants

For the present study, a total of 142 fully vaccinated individuals were consecutively enrolled in a routine care setting at 6 ± 1 weeks after their second vaccination. In a prospective single-centre, cross-sectional study design, these patients were recruited in the rheumatological outpatient clinic of MED|BAYERN OST, Medizinische Versorgungszentren Altötting Burghausen, Burghausen, Germany. The total study sample consisted of two independent subgroups. Eighty patients with rheumatoid arthritis received MTX in monotherapy as a disease-modifying antirheumatic drug (DMARD). 15 of 80 patients (18.8%) additionally received prednisolone with a mean dosage of 3.88 mg/die. The comparison control group without MTX medication comprised 62 patients diagnosed with osteoarthritis of the hands and not undergoing treatment with immunomodulatory medication.

Our primary goal in this study was to compare the humoral immune responses between independent groups of patients with and without MTX medication when vaccinated against SARS-CoV-2 based on neutralising antibody titres 6 ± 1 weeks after the second vaccination with vaccines from BioNTech/Pfizer, Moderna, and AstraZeneca.

All patients provided written informed consent for study participation and publication of the scientific data obtained. This study was approved by the ethics committee of the University Hospital of Würzburg, Germany. The process of organising and conducting the study followed the principles of "Good Clinical Practice" [11, 12].

The primary inclusion criterion for the MTX subgroup was a confirmed diagnosis of RA according to ACR-EULAR 2010 criteria. Further criteria for study participation were at least 18 years of age and the presence of a signed informed consent form.

The exclusion criteria were a relative or absolute contraindication for therapy with MTX, a previously known intolerance of MTX, and a history of SARS-CoV-2 infection.

The comparison group consisted of individuals without inflammatory rheumatic disease and not undergoing DMARD therapy, e.g. patients with osteoarthritis of the hands.

### Measurements of immune response

Our primary endpoint in this study was to measure the immune response as evaluated by titres of neutralising antibodies against SARS-CoV-2. These titres were determined using a quantitative ELISA test for the IgG antibodies against the S1 antigen of SARS-CoV-2: Anti-SARS-CoV-2-QuantiVac ELISA (IgG); manufacturer: EUROIMMUN Medizinische Labordiagnostika AG, Lübeck, Germany.

### Statistical analysis

Sample size considerations referred to the primary study analysis (comparing vaccination responses in patients with and without MTX medication). Therefore, the sample size calculation assumed two independent samples, a significance level of 5% and statistical power of at least 80%, to detect a medium effect size (*d* = 0.5). Based on this background, the optimal sample size for one-sided testing was calculated to be a total of 102 subjects (i.e. at least 51 individuals per independent study subgroup: MTX patients vs. controls).

Data management and statistical analyses were performed for all data as appropriate using Microsoft Excel or SPSS (German version 17.0.0) software, respectively [13]. All inferential tests were considered to be statistically significant at *P* < 0.05. Pearson’s chi-square tests were used to compare frequencies of categorical variables between patient subgroups. Moreover, an analysis of variance (one-way ANOVA) was performed to test for mean differences in continuous variables between both described independent patient subgroups with and without MTX medication. For the primary outcome measure (antibody titres), we used Mann–Whitney *U* tests to test for differences in central tendency between subgroups with and without MTX medication. This was because the distribution of titres of neutralising antibodies were analysed and found to reach only ordinal data levels due to lab-related ceiling effects.

## Results

Between April 12, 2021, and September 14, 2021, the patients of both included study arms (RA with MTX vs. controls) were recruited and showed up for study participation.

The total sample of the present study was *n* = 142 patients. It was composed of an MTX treatment subgroup (80 patients, 56.3%) and a reference group (62 patients, 43.7%). The time of blood sampling—and thus evaluation of antibody titres—was 6 ± 1 weeks for all included study subjects.

The main characteristics of both subgroups are displayed in Tables 1 and 2. The most remarkable group difference was the significantly higher age of patients in the MTX group with rheumatoid arthritis (mean age of 71.9 years vs. 64.3 years in the reference group; this group difference was most striking and statistically significant: *P* < 0.001). Moreover, the percentage of female patients was higher in the subgroup of individuals treated with methotrexate (79.0% vs. 61.3%; *P* = 0.023).

**Table 1:** Patient characteristics, including relevant medical data (stratified by patient subgroup)

	MTX-exposed RA patients (*n* = 80)	Reference group without MTX (*n* = 62)	*P* value
Age (years)	71.9	64.3	< 0.001
Female sex (%)	79.0	61.3	
Male sex (%)	21.0	38.7	0.023
Mean RA disease duration (years)	8.53	0	n.a
Mean dose MTX (mg/week)	13.44	0	n.a
Seropositivity (%)	86.3	0	n.a
Prednisolone use (%)	18.8	0	n.a
Mean dose prednisolone (mg/day)	3.88	0	n.a
Diabetes (%)	18.8	12.9	0.348
Mean GFR values (mL/min)	75.46	81.26	0.034
Mean RR syst. (mm Hg)	141.81	143.00	0.871
Mean RR diast. (mm Hg)	78.95	83.44	0.316
Overall tolerability (sec. vacc.)	1.58	1.79	0.205
BioNTech/Pfizer (%)	72.5	67.7	
AstraZeneca (%)	18.8	22.6	
Moderna (%)	8.8	9.7	0.820
SARS-CoV-2 IgG (BAU/mL)	309.0	345.1	0.040
Maximum response (≥ 384 BAU/mL) (%)	63.8	77.4	
Moderate response (176–383 BAU/mL) (%)	12.5	14.5	
Low response (34–175 BAU/mL) (%)	20	8.1	
Nonresponse (< 34 BAU/mL) (%)	3.8	0	0.039

**Table 2:** Sociodemographic and medical data within MTX group (stratified by age subgroup)

	Age < 55 (*n* = 9)	Age 55–70 (*n* = 17)	Age > 70 (*n* = 54)	*P* value
Age (years)	49.8	64.8	77.8	< 0.001
Female sex (%)	77.8	70.6	55.6	
Male sex (%)	22.2	29.4	44.4	0.301
Mean RA disease duration (years)	7.8	9.6	8.3	0.770
Mean dose MTX (mg/week)	15.8	14.2	12.9	0.137
Seropositivity (%)	88.9	76.5	88.9	0.419
Prednisolone use (%)	0	29.4	18.5	0.188
Mean dose prednisolone (mg/day)	0	4.3	3.7	0.539
Diabetes (%)	0	0	27.8	0.012
Mean GFR values (mL/min)	93.3	83.2	70.1	< 0.001
Mean RR syst. (mm Hg)	158.0	127.8	143.9	0.045
Mean RR diast. (mm Hg)	95.7	77.9	77.6	0.035
Overall tolerability (sec. vacc.)	2.3	1.5	1.5	0.140
BioNTech/Pfizer (%)	88.9	70.6	70.4	
AstraZeneca (%)	0	17.6	22.2	
Moderna (%)	11.1	11.8	7.4	0.598
SARS-CoV-2 IgG (BAU/mL)	384.0	347.5	284.4	0.025
Max. response (≥ 384 BAU/mL) (%)	100	76.4	53.7	
Moderate response (176 to 383 BAU/mL) (%)	0	11.8	14.8	
Low response (34 to 175 BAU/mL) (%)	0	11.8	25.9	
Nonresponse (< 34 BAU/mL) (%)	0	0	5.6	0.159

Notably, the frequencies of used vaccines were comparable and did not differ significantly in either study cohort (see Table 1). The vaccines used were distributed in the total sample as follows: BioNTech/Pfizer (70.4%), AstraZeneca (20.4%), and Moderna (9.2%).

Most noticeably, MTX-exposed study patients were significantly older than individuals in the reference group (*P* < 0.001; see above). Therefore, this covariate was especially considered and analysed in detail when performing subsequent statistical analyses.

Importantly, laboratory data regarding neutralising SARS-CoV-2 antibodies turned out to provide ceiling effects in a good deal of participating individuals: A total of 99 out of 142 study participants (69.7%) showed a maximum antibody response of ≥ 384.0 BAU/mL, representing the upper limit of the measurement range of the test. Considering this point, we decided to statistically analyse the response to vaccination using nonparametric procedures [ordinal data level of evaluated antibody titres—4-point Likert scale: nonresponse (< 34 BAU/mL), low (34–175 BAU/mL), moderate (176–383 BAU/mL), and maximum vaccine response (≥ 384 BAU/mL)].

Overall comparison of the central tendency of evaluated antibody titres based on ordinal data levels between both study subgroups demonstrated that MTX-treated individuals showed a lower antibody response to SARS-CoV-2 vaccination (*P* = 0.039; Mann–Whitney *U* test, see Table 1); 63.8% of MTX-treated patients showed a maximum response, whereas 77.4% of controls were complete responders. This result, however, does not yet consider the influence of age as a covariate and must therefore not be interpreted merely as displayed. To consider that MTX patients were significantly older (see above), which presumably affects immunogenicity to a relevant deal, we performed ordinal regression analyses, simultaneously including MTX medication *and* age as predictor variables. This analysis, aiming at the determination of the influence of age on immunogenicity, revealed that—considering all 142 study subjects—neutralising antibody titres after the second vaccination were best and significantly predicted by the variable age (*P* = 0.005). In contrast, MTX did not significantly predict the measured immune response (*P* = 0.365). Therefore, regarding the total study sample, age is the most significant and clinically relevant predictor of vaccination success as measured by neutralising antibody titres.

Interestingly, the negative correlation between age and immunogenicity was more pronounced and statistically significant in the subgroup of patients with rheumatoid arthritis and MTX treatment (*r* = − 0.293; *P* = 0.008) than in controls (*r* = − 0.124; *P* = 0.335). To further investigate this observation, we evaluated the response in different age subgroups of our study cohort. Thus, we were able to check for possible interactions between patient age and MTX medication with respect to vaccination response as measured by neutralising antibody titres:

To control for the influence of the variable age, we established three age-based subgroups (< 56 years; 56–70 years; > 70 years) within our total study cohort and compared the measured immune responses between study participants with and without methotrexate medication.

Whereas titres of neutralising antibodies were not significantly different between MTX and non-MTX patients in patients less than 70 years of age (< 56 years: *P* = 0.234; 56–70 years: *P* = 0.446), MTX patients showed a significantly reduced immune response to vaccination in the oldest patient subgroup (> 70 years: *P* = 0.038). In these (oldest) patients, non-MTX patients showed the maximum immune response in 76.5% of cases—the corresponding percentage was reduced to 53.7% in study participants on MTX medication (effect size *d* = 0.21).

**Fig. 1:**
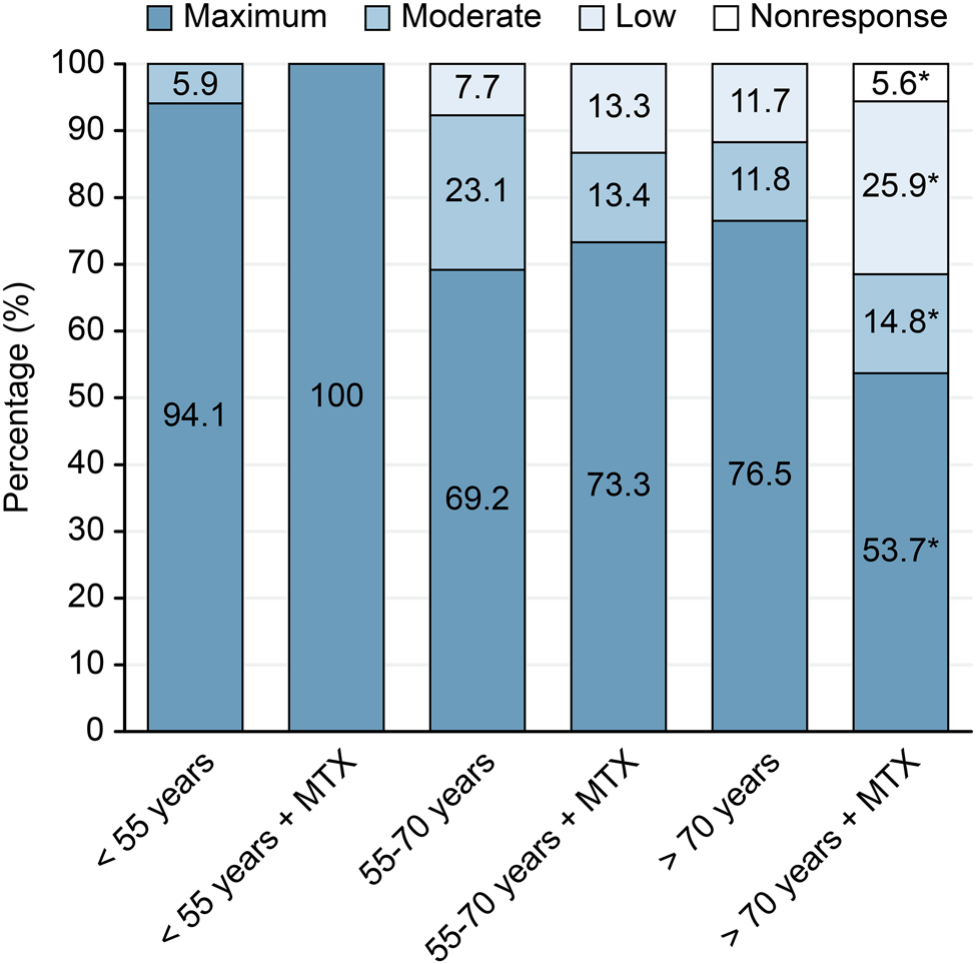
The humoral immune response measured by titres of neutralising IgG antibodies against S1 antigen of SARS-CoV-2 in dependence on age and use of methotrexate (4-point Likert scale: nonresponse (< 34 BAU/mL), low (34–175 BAU/mL), moderate (176–383 BAU/mL), and maximum response (≥ 384 BAU/mL). Immuno-response in terms of titres of neutralising antibodies differs significantly between MTX and non-MTX patients only for patients > 70 years (*P* = 0.038; *d* = 0.21). Sample sizes of the subsamples: < 55 years (*n* = 17); < 55 years + MTX (*n* = 9); 55–70 years (*n* = 28); 55–70 years + MTX (*n* = 17); > 70 years (*n* = 17); > 70 years + MTX (*n* = 54)

This last evaluation shows that, based on our data, the combination of MTX administration and older age significantly impairs the humoral immune response (Fig. 1).

Glucocorticoid use did not effect on vaccine response in our cohort. This was due to the low mean daily dose of 3.88 mg prednisolone. A total of 81.2% of patients had no therapy with glucocorticoids. The mean dose of methotrexate per week decreased depending on age: 15.83 vs. 14.22 vs. 12.87 mg (data not shown). The differences were not statistically significant. The MTX dose decreasing with age was likely due to the renal function impairment frequently observed at higher ages.

## Discussion

Numerous papers on vaccination against SARS-CoV-2 have been published or are available as preprints. An increasing number of these studies are addressing the issue of immunogenicity under immunomodulatory or immunosuppressive therapy. Based on experiences referring to attenuated vaccination responses against influenza and pneumococcus under immunomodulatory agents [10], there was great uncertainty at the beginning of the COVID vaccination campaigns amongst both physicians and patients [4].

The first studies on this topic were conducted by Geisen et al. [14] and Furer et al. [15]. In a monocentric study involving 26 patients with inflammatory rheumatic diseases, Geisen et al. [14] showed that vaccination with mRNA-based vaccines led to seroconversion seven days after the second vaccination, albeit at a reduced rate compared with healthy controls. The multicentre observational study by Furer et al. [15] evaluated the immunogenicity and safety of the BioNTech/Pfizer mRNA vaccine (BNT162b2) in adult patients with an inflammatory rheumatic disease (*n* = 686) compared with the general population (*n* = 121). IgG antibody levels against SARS-CoV-2 spike S1/S2 proteins were measured 2–6 weeks after the second vaccination. The proportion of vaccinated individuals with seroconversion (defined as IgG ≥ 15 BAU/mL) was significantly reduced in patients with the inflammatory rheumatic disease compared to healthy controls (86% (*n* = 590) vs. 100%, *P* < 0.0001). Treatment with glucocorticoids, rituximab, mycophenolate mofetil, and abatacept was a major risk factor for reduced vaccine response. Amongst patients on rituximab, seropositivity defined as S1/S2 IgG ≥ 15 BAU/mL was detectable in only 39% of cases. Thus, the risk for a lack of seroconversion was highest in these patients, which is not surprising given the B cell depleting properties of rituximab. However, a reduced rate of seroconversion was also observed for methotrexate, the conventional synthetic disease-modifying antirheumatic drug (csDMARD) most commonly used worldwide in rheumatoid arthritis. However, the effect, in this case, was only mild. Interestingly, older age was a risk factor for an attenuated vaccination response to SARS-CoV-2 in this work. These initial findings by Geisen et al. [14] and Furer et al. [15] could essentially be confirmed by other research groups [2, 5, 16, 17]. Whereas bDMARDs directed against cytokines, such as anti-TNFalpha or anti-IL-17, are associated with only a little or no attenuation of the humoral vaccine response, this type of immune response seems to be reduced in the case of biologics directed against cells, such as rituximab or abatacept, but also in the case of csDMARDs, such as mycophenolate mofetil, methotrexate or prednisolone [3, 18, 19]. The addition of MTX to a bDMARD directed against cytokines also attenuated the humoral response compared to monotherapy with bDMARDs [3]. In another study by Simon et al., it was shown that patients with systemic inflammatory diseases generally, regardless of whether tsDMARD, bDMARD, csDMARD, or even no immunomodulatory therapy was administered, developed a delayed and overall reduced humoral vaccine response compared with controls [20]. In contrast, a recently published paper, although demonstrating the overall reduced humoral response in immune-mediated inflammatory diseases (IMID), did not find a statistically significant impact on the humoral response for age, gender, underlying rheumatic disease, or for treatment with glucocorticoids, bDMARDs, or methotrexate [21]. Rather, this publication found evidence that viral vector vaccines are associated with a poorer humoral response than mRNA vaccines in IMID patients. Overall, the data available concerning the determinants of seroconversion after vaccination against SARS-CoV-2 remain inconsistent and partly contradictory to date. In particular, the data of Manolache et al. [21] are partly contradictory to previous publications and our data. This may be due to the heterogeneous patient population, the heterogeneous DMARD therapies applied, and the study’s low number of cases.

Tzioufas et al. studied the effect of modifications of DMARD therapy during the vaccination phase [17]. They found that extensive suspension of DMARD therapy [22] resulted in equalisation of vaccination response between patients with inflammatory rheumatic diseases and healthy controls, again underscoring the influence of therapy. Discontinuation of the medication for several weeks before and after vaccination, as applied here, is problematic in controlling the underlying disease and, based on our data, not at all necessary concerning MTX in younger and middle-aged patients. Boekel et al. [23] showed that older patients on DMARD therapy had a reduced humoral response at the time of vaccination, especially after the first vaccination with a vector or mRNA vaccine. The authors concluded that early vaccination with the second dose should be performed, especially in elderly patients on immunosuppressive therapy.

This and further studies served as the basis for recommendations by various scientific societies on the management of DMARD therapy in patients with inflammatory rheumatic diseases. For example, the German Society for Rheumatology (DGRh) recommended the third vaccination against SARS-CoV-2 early on for patients who are under ongoing therapy with rituximab (vaccination within one year after the last RTX administration), cyclophosphamide, abatacept, MMF, or higher-dose glucocorticoids (> 20 mg prednisolone equivalent/die) following individual risk assessment as early as four weeks after completion of baseline immunisation. Regarding MTX, the DGRh differs based on the dose applied. At doses of < 20 mg/week, no relevant attenuation is assumed, unlike doses of > 20 mg/week. Especially at the latter dosage, pausing MTX for two weeks after vaccination might be considered. Age was not considered in these recommendations, although based on our data, MTX led to an attenuation of the humoral response only in patients of > 70 years of age; thus, a cessation would be worth discussing only in this age group. The EULAR also gives recommendations for the vaccination of patients with inflammatory rheumatic diseases and lists methotrexate as possibly having a mild attenuative effect on humoral vaccination response [24]. The American College of Rheumatology also recommends discussing an undefined interruption of DMARD therapy with the patient in the case of a stable phase of the disease. However, the panel of experts could not achieve agreement on the extent to which this also applies to bDMARDs directed against cytokines, such as TNFalpha, IL-17, IL-1R, IL-6R, IL-23, or IL-12/23 [25]. Hence, the EULAR and ACR recommendations also do not explicitly consider the age at vaccination [26]. Nomura et al. [27] demonstrated that elderly patients with SARS-CoV-2 show a significant and rapid decrease in antibody levels between three and six months after two vaccinations with an mRNA-based vaccine. Furthermore, advanced age is considered a major risk factor for symptomatic infection with SARS-CoV-2, hospitalisation or the need for intensive care, or even death, despite two vaccinations [28]. In the light of this, age appears to be highly relevant, especially concerning a recommendation to suspend DMARD therapy with methotrexate. According to our data, an interruption of methotrexate medication would not be necessary for younger and middle-aged patients with rheumatoid arthritis. We could not show a dose-dependent effect of MTX or an attenuation of the vaccination response in the ≤ 70-year-old group. Only in the group of > 70-year-old patients did MTX result in a statistically significant decrease across all four response categories. Doses above 20 mg, as explicitly mentioned in the recommendation of DGRh and EULAR, were not used in our cohort. In our collective, fortunately, no difference in the tolerability of the vaccination was found between patients with rheumatoid arthritis and methotrexate compared to controls (data not shown). Our results are thus congruent with previously published data [4, 14, 15]. The reported symptoms were consistent with the expected vaccination reactions in terms of severity and frequency. Confirmed relapses in rheumatoid arthritis were not seen in our setting (data not shown).

The main limitations of our study are the lack of data on the cellular vaccine response and the use of different vaccines. Current studies do not necessarily indicate reduced cellular immunity due to a reduced humoral vaccination response. For example, in the context of rituximab and the associated depletion of CD20-positive B lymphocytes, T cellular immunity was detectable in patients with inflammatory rheumatic diseases despite the absence of seroconversion [19]. Similarly, reduced seroconversion was not associated with a reduced T cell response in a cohort of patients with psoriasis vulgaris taking methotrexate [29].

In summary, older age in patients with rheumatoid arthritis combined with methotrexate results in a significantly reduced humoral response after vaccination against SARS-CoV-2. Methotrexate alone or rheumatoid arthritis itself, in contrast, had no significant effect on the level of neutralising antibodies to the spike protein in our study. These data underline the importance of age on the humoral response against SARS-CoV-2 and once again identify elderly patients as a particularly at-risk group. Therefore, our results may support the temporary cessation of methotrexate, particularly in patients above 70 years of age, in the context of vaccination against SARS-CoV-2. Prospective and longitudinal studies in larger cohorts are needed to confirm the serologic findings using tailored clinical endpoints, such as the prevention of infections.

## Data Availability

The data underlying this article will be shared upon reasonable request to the corresponding author.

## Code availability

Data management and statistical analyses were performed for all data as appropriate using Microsoft Excel or SPSS (German version 17.0.0) software, respectively.
